# Effects of Roy's Adaptation Model on Quality of Life in People with Opioid Abuse under Methadone Maintenance Treatment: A Randomized Trial

**DOI:** 10.4314/ejhs.v33i2.21

**Published:** 2023-03

**Authors:** Meysam Rezazadeh, Seyyed Abbas Hosseini, Amir Musarezaie

**Affiliations:** 1 M.Sc. Student of Nursing, Student Research Committee, Isfahan University of Medical Sciences, Isfahan, Iran; 2 Department of Adult Health Nursing, Isfahan University of Medical Sciences, Isfahan, Iran

**Keywords:** Nursing Model, Quality of Life, Opioid Abuse, Methadone, Nursing

## Abstract

**Background:**

Opioid abuse is one of the most obvious problems in today's world and directly affects individuals' quality of life. The present study aimed to investigate the effects of Roy's adaptation model on the quality of life in people with opioid abuse under methadone maintenance treatment.

**Methods:**

This randomized trial study was conducted in 2021 on 72 patients with opioid abuse under methadone maintenance treatment at the Center for Addiction Harm Reduction in Isfahan. The samples were randomly allocated into intervention (n=36) and control groups (n=36) based on the table of random numbers by computer. The intervention was conducted by implementing Roy's adaptation model in the intervention group. To analyze the data, paired t-test, independent sample t-test, chi-square test, and analysis of covariance were used.

**Results:**

The mean ± standard deviation of the quality of life score in the intervention group (28.96±4.79) was significantly different than before the intervention (24.02±6.09) (P<0.001). At the same time, it was not significantly different in the control group. The mean ± standard deviation of the quality of life score in the intervention group (24.02±6.09) was not significantly different from the control group (20.55±8.53) before the intervention.

**Conclusion:**

Roy's adaptation model had positive effects on the quality of life score in patients with opioid abuse. On the other hand, patients' quality of life indicates the effectiveness of methadone maintenance treatment. Therefore, it is suggested to use this model in nursing care programs.

## Introduction

Statistics of the United Nations World Drug Report, more than 35 million people worldwide had substance abuse disorders and needed medical care in 2019, while only one in seven had access to treatment (1). Iran is also one of the most important countries with addiction problems and according to the latest official statistics, there are one million opioid users, including substance abuse and recreational use in this country (2).

In studies on the effects of substance abuse on quality of life (QOL), it has been indicated that the QOL in patients with opioid abuse reduces in different stages of disease and recovery, either from a subjective or objective perspective, in different modes. In addition, the evaluation of the QOL process can be used to determine the effect of various medical and non-medical measures (3). In this regard, nurses can create a relative adaptation in patients with opioid abuse and play a key role in their QOL through structured care and using nursing theories (4, 5). Nurses need a nursing model to perform structured care because the nursing models act as care guidelines (6).

Roy's adaptation model (RAM) has been used in nursing research, education, and care for many years (7). It improves the person's interaction with the environment and causes better adaptation (8). Adaptation through RAM is achieved by reducing incompatible behaviors and changing such behaviors to adaptive behaviors (7, 9). RAM-based nursing care is formed within the nursing process (10). Individuals' participation in the process of care and treatment increases the quality of nursing care and health recovery (10). In this regard, RAM emphasizes the participatory nature of the treatment program and patients' involvement in planning (11). This leads to patients' independence on the treatment team and supports patients' rights to participation and involvement in care (12). In RAM, nurses continuously communicate with groups, families, and people to evaluate their adaptation, and help them to cope with health problems (13).

The studies that used RAM for nursing care in chronic diseases such as chronic obstructive pulmonary disease and heart failure disease showed positive results of the model, such as increasing adaptation and QOL (10, 14). As stated, compliance with and continuing the pharmacological and non-pharmacological treatment of addiction can be one of the important causes of improving the QOL in patients with opioid abuse, as indicated in the study of Cao et al. (15). Moreover, beginning and continuing treatment through nursing intervention and care can lead to the continuation of pharmacological and non-pharmacological treatment, resulting in increased QOL in patients with opioid abuse (5). However, there are still shortcomings in creating stability and continuation of pharmacological and non-pharmacological care, which can be due to a lack of attention to all modes of maladaptation in such patients.

While several studies have been conducted on the effectiveness of different types of psychological therapies on patients with opioid abuse (3, 16), there is no study on the effectiveness of RAM in improving their QOL. Therefore, this study aimed to investigate the effects of RAM on the QOL in people with opioid abuse under methadone maintenance treatment (MMT).

## Methods

This randomized trial as a parallel study with an allocation ratio (1:1) was conducted for three months from December 22, 2021, to March 20, 2022, on 72 people including intervention (n=36) and control groups (n=36) with opioid abuse under MMT at the Center for Addiction Harm Reduction in Isfahan, Iran.

To provide the intervention for the participants in the control group, the researcher undertook to provide it to them after evaluating the effectiveness of RAM. However, none of the participants were informed of their allocated group and the control group received the routine intervention and did not know about the main intervention. All of the participants were informed of their rights to participate if they are willing, to withdraw at any time and that their information would be confidential, and they incur no charge for participating in the study. All participants signed the written informed consent form and their rights were respected under the Helsinki Declaration.

The intervention based on RAM in the intervention group was performed by the researcher (nurse) in four modes physiologic, self-concept, role function, and interdependence based on observation, interview, and measurement. Then, the focal, contextual, and residual stimuli associated with incompatible behaviors were identified. While no intervention was done in the control group and they only received routine interventions, in the intervention group, the interventions within the nursing process were performed as follows:

1. Determining standard nursing diagnoses: Include incompatible behaviors and behaviors that may be incompatible in the future.

2. Planning to set the goals: In this stage, goals were determined based on requirements. Then, the nursing care required for achieving goals was determined. Nursing care included interventions in which the focal, contextual, and residual stimuli altered or were modified until the focal stimulus led the patients to adapt.

3. Implementation: In this stage, the researcher acted as an external moderator to expand the range of adaptation and establish an adaptive behavior. The researcher changed or modified the stimuli or conditions rather than individuals and thus, the interaction of individuals with the environment improved, and their health was maintained. In this stage, group or individual interventions were performed based on patients' needs for three months.

4. Evaluation: The researcher re-evaluated the predetermined goals and plans. The researcher examined the achievement of goals and determined the adaptation level in health and disease modes.

**Figure 1:**
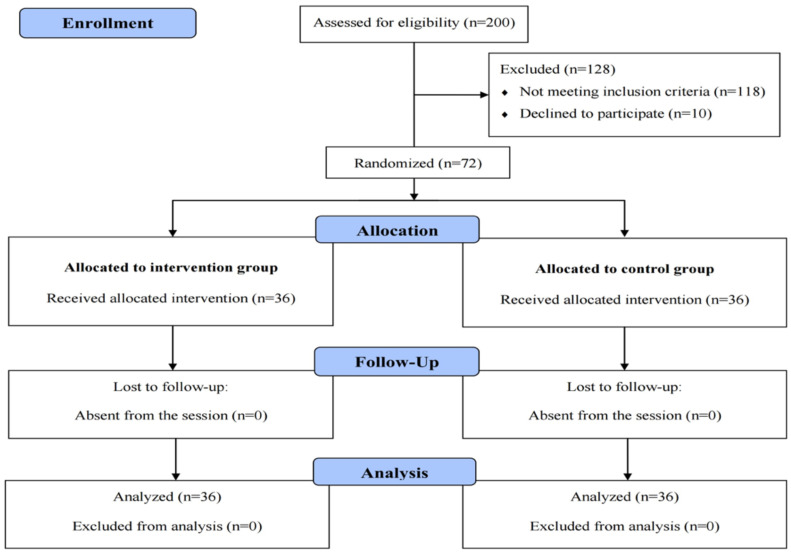
Flow chart of the study

And at the end of the intervention (end of three months), the QOL in the intervention and control groups was re-evaluated by a self-report questionnaire and the changes were compared with that obtained before the intervention. Considering the study of Soleimani et al. in 2017 and the 10% of sample loss, the sample size was estimated as 72 participants according to the sample size formula (n=[Z_1_+Z_2_]^2^[2S^2^]/d^2^, Z_1_=1.26, Z_2_=0.8, S=0.7, d=0.7S) with the test power of 80% (17). The inclusion criteria were the age range of 18-65 years old, literacy, absence of severe physical, mental, and psychological diseases, and interest and willingness to participate in the study. The exclusion criteria were the exclusion from the study during the intervention for any reason and the death of the participant during the study. The samples were randomly (simple randomization) allocated into intervention (n=36) and control groups (n=36) based on the table of random numbers by computer. In such a way the researcher read the numbers in a top-down fashion, and even numbers were assigned to the intervention group and odd numbers to the control group then assignments were numbered in sealed envelopes (Figure 1).

To collect data, a two-part instrument was used. The first part included demographic characteristics including age, age of onset of consumption, employment status, education level, and marital status, which were completed in the self-report form by the participants. The second part was the Persian version of the short-form 36-item QOL questionnaire (SF-36) was employed for data collection (18). The SF-36 is a valid and reliable questionnaire that contains 36 questions and assesses QOL in eight domains including ten items on physical functioning, seven items on role function, two items on physical pains, five items on general health perception, four items on vitality, two items on social function, one item on emotional function, and five items on mental health. Three-, five- and six-choice questions of the SF-36 are scored on three-, five-, and six-point scales of ‘0, 50 and 100’, ‘0, 25, 50, 75, and 100’, and ‘0, 20, 40, 60, 80, and 100’, respectively. Therefore, the total score of each domain ranges from 0 to 100, the higher the score the better the QOL (16). This instrument was completed by the participant in a self-report form. To estimate the validity, Montazeri et al. used convergent validity and the comparison of cognitive groups on 4163 participants in the age group of 14 years and above. The reliability of the questionnaire ranged between 0.77 and 0.90, and its validity ranged between 0.58 and 0.95 using the convergence validity test (18).

The data were analyzed by SPSS 26 (USA, IL, Chicago, Inc., SPSS), and descriptive methods such as frequency, percentage, mean, and standard deviation (SD) were used to describe the data. The normality of the quantitative variables was evaluated using the Kolmogorov-Smirnov test and the normal distribution was confirmed. To compare the two groups in terms of the qualitative variables, the chi-square test and paired t-test were used for intragroup comparison. The independent sample t-test was used to compare the two groups before the intervention, and analysis of covariance (ANCOVA) was used for intragroup comparison after the intervention. The significance level was determined as <0.05 in all tests.

**Ethical consideration:** This research was approved by the Research Ethics Committee of Isfahan University of Medical Sciences (ethics code: IR.MUI.NUREMA.REC.1400.159, project No. 3400574) and was registered in the Iranian Clinical Trials Registry (code: IRCT20211121053121N1).

## Results

Of the 72 participants in the study, 36 participants in the intervention group and 36 participants in the control group were present at the end of the intervention. The participants' age means ± SD in the intervention and control groups was (39.00±10.12) and (43.19±8.27), respectively, which according to the independent sample t-test was not significant (*P*>0.05; Table 1). The employment status, education level, and marital status obtained by the chi-square test were not significant (*P*>0.05; Table 1). The age of onset of consumption as an important variable in the QOL was significant (*P*<0.05; Table 1) that considered a confounding variable and its effect was adjusted by ANCOVA (Table 1).

**Table 1:** Comparison of the study groups in terms of participants' demographic characteristics

Characteristics	Groups^a^		*P*-value
		
	Intervention (n=36)	Control (n=36)	
**Age (years)**	39.00 ± 10.12	43.19 ± 8.27	0.058^b^
**Age of onset of consumption**			0.039^c^
Over 18 years	31 (86.10)	24 (66.70)	
Under 18 years	5 (13.90)	12 (33.30)	
**Employment status**			0.059^c^
Freelance job	23 (63.90)	15 (41.70)	
Unemployed	13 (36.10)	21 (58.30)	
**Education level**			0.135^c^
Primary education	9 (25.00)	13 (36.10)	
Intermediate education	11 (30.60)	15 (41.70)	
Diploma and College Education	16 (44.40)	8 (22.20)	
**Marital status**			0.380^c^
Single	13 (36.10)	16 (44.40)	
Married	11 (30.60)	6 (16.70)	
Separation or Divorced	12 (33.30)	14 (38.90)	

a Values are presented as mean ± SD or n (%)

b The results of the independent sample t-test

c The results of the Chisquare test

The mean ± SD of the QOL score in the intervention group (December 22, 2021, to March 20, 2022) (28.96±4.79) was significantly different from that obtained before the intervention (24.02±6.09) using the paired t-test (*P*<0.001) while it was not significant in the control group (*P*>0.05, Table 2). The mean ± SD of the QOL score in the intervention group (24.02±6.09) was not significantly different from the control group (December 22, 2021) (20.55±8.53) using the independent sample t-test (*P*>0.05) while after the intervention (March 20, 2022), ANCOVA showed that it was significantly different between the two groups (28.96±4.79 vs. 20.27±6.61) (*P*<0.001, Table 2). The nature of the study was such that it did not have any side effects or harm to the study participants.

**Table 2:** Within and between-group comparisons in terms of the mean score of the QOL

Groups^a^	Time		*P*-value
		
	Before intervention (n=36)	After intervention (n=36)	
**Intervention**	24.02 ± 6.09	28.96 ± 4.79	0.000^c^
**Control**	20.55 ± 8.53	20.27 ± 6.61	0.751^c^
***P*-value**	0.051^b^	0.000^d^	

a Values are presented as mean ± SD

b The results of the independent sample t-test

c The results of the paired sample t-test

d The results of the ANCOVA.

## Discussion

The QOL scores of the patients under MMT were higher than in previous studies (19). This difference may be due to the lower age mean of the participants in the present study (39 years). Previous studies indicated that, apart from the medical costs of MMT, there was a negative relationship between older age and QOL (20). Other studies showed that work had positive effects on drug users' mental health; that is, working people had better mental health (21). In addition, it was indicated that educated people who used drugs were more likely to be affected by mental disorders, but the relationship was not significant (21). In this study, the two groups were not significantly different in terms of education. Sa'adati et al indicated that the two groups of industrial and traditional drug users were not significantly different in terms of occupation and education, but the average income of the two groups was different, and industrial drug users had higher incomes, which could be one of the reasons for a better QOL and mental health (22). In line with a study in Taiwan, the present study showed that married patients had higher QOL than other groups (23). The married patients were supported by their spouses not only for their daily activities but also for MMT. Their spouses supported them emotionally and encouraged them to worry less and deal with fewer psychological problems, and as a result, have a better QOL.

Previous studies indicated considerable changes in QOL in people with opioid abuse under MMT (24). However, some studies showed no significant results (25). On the other hand, improved QOL after addiction treatment was proved to be associated with reduced substance abuse (26). The drugs evaluated in the study of Sa'adati et al. were heroin, amphetamine, and opium; however, the type of drug was not associated with any aspect of QOL (22). Longer MMT contributed to a better QOL, as confirmed by some other studies (19, 24).

Roy has suggested that patients' physical, social, cultural, spiritual, and intellectual needs should be met to ensure that they would be adapted to any condition (27). Roy believed that reciprocal attachment between family, relatives, friends, and other support systems affects the patient and his/her relationships (28).

Since patients' cooperation in care-related intervention, as teamwork with nurses and other specialists, is an effective step in controlling disease, the present study showed significant changes in patients' adaptation during three months of intervention to meet their needs with the coordination of nurses and their support. Since nursing care has a holistic approach, training patients and their families can significantly affect their self-efficacy and QOL.

Considering human, financial, and energy constraints, the present study faces some limitations. On the one hand, the purpose of the study was limited to patients with opioid abuse under MMT at the Center for Addiction Harm Reduction. Sample size and patient differences prevent the generalization of the results. Further intervention groups and studies are required to observe the effects of the intervention and confirm the results. In addition, due to time limitations, the present study evaluated the effects of the intervention for three months which is relatively short, and continuous follow-up is required to determine the long-term effects of the adaptation intervention.

The results of the present study have important consequences for MMT for health and medical centers because the patients' QOL is an indicator of the effectiveness of MMT that should be consistently evaluated during treatment. Research on providing services, especially nursing care, should be most concentrated on consistency and continuation of treatment for patients who receive MMT as well as the relationship between these factors with QOL to develop models for improving QOL and continuing their treatment.
